# Decreased risk of chronic fatigue syndrome following influenza vaccine: a 20-year population-based retrospective study

**DOI:** 10.1186/s12967-025-06600-5

**Published:** 2025-07-10

**Authors:** Hsun Chang, Wei-Cheng Yao, Teng-Shun Yu, Heng-Jun Lin, Fuu-Jen Tsai, Shinn-Ying Ho, Chien-Feng Kuo, Shin-Yi Tsai

**Affiliations:** 1 Division of Infectious Diseases, Department of Internal Medicine, MacKay Memorial Hospital, Taipei, Taiwan; 2 Department of Medicine, MacKay Medical College, New Taipei City, Taiwan; 3 Department of Anesthesiology and Pain Medicine, Min-Sheng General Hospital, Tao-Yuan, 330 Taiwan; 4 Management Office for Health Data, China Medical University Hospital, China Medical University, Taichung, 404327 Taiwan; 5 Department of Internal Medicine, Taichung Veterans General Hospital, Taichung, Taiwan; 6 School of Chinese Medicine, College of Chinese Medicine, China Medical University, Taichung, 404328 Taiwan; 7 Department of Medical Research, China Medical University Hospital, China Medical University, Taichung, 404327 Taiwan; 8 Institute of Bioinformatics and Systems Biology, National Yang Ming Chiao Tung University, Hsinchu, Taiwan; 9 Biomedical Engineering, National Yang Ming Chiao Tung University, Hsinchu, Taiwan; 10 Department of Nursing, Nursing and Management, MacKay Junior College of Medicine, New Taipei City, 25245 Taiwan; 11 Institute of Biomedical Sciences, MacKay Medical College, New Taipei, Taiwan; 12 Department of Laboratory Medicine, MacKay Memorial Hospital, Taipei City, Taiwan; 13 Department of Health Policy and Management, Johns Hopkins Bloomberg School of Public Health, Johns Hopkins University, Baltimore, MD USA; 14 Department of Medical Laboratory and Regenerative Medicine, Mackay Medical College, New Taipei City, Taiwan

**Keywords:** Chronic fatigue syndrome (CFS), Influenza, Preventive medicine, Vaccination, Population-based study, Long-term complication, Burden

## Abstract

**Background:**

Chronic Fatigue Syndrome (CFS) is a debilitating condition often follows infections, including influenza. Influenza frequently results in fatigue during the acute stage. However, the data regarding the association of influenza, vaccine and CFS is scarce. Thus, this study aims to investigate whether influenza increases the risk of developing CFS and examine the impact of influenza vaccination and severity of influenza on this risk.

**Methods:**

We conducted a national, population-based cohort study, using data from the National Health Insurance Research Database (NHIRD) of Taiwan, which identified 309,692 patients aged 20 years or older who were newly diagnosed with influenza between 2000 and 2019. An equal number of participants without influenza were also identified. Both groups were followed up until the end of CFS diagnoses. Cox proportional hazards regression analysis was used to calculate adjusted hazard ratios(aHRs) and 95% confidence intervals (CIs) for CFS as associated with influenza, adjusting for demographic factors and comorbidities. We also evaluated the effects of influenza vaccination and severe influenza.

**Results:**

After propensity matching, each cohort comprised 309,692 patients. Over an average follow-up period of approximately 12 years, influenza patients exhibited a significantly increased risk of developing CFS compared to matched controls (aHR = 1.51; 95% CI: 1.48–1.55; *p* < 0.001). The increased risk of CFS among patients with influenza was consistent across all age groups and both sexes, with the most pronounced elevation observed in older individuals. Patients who experienced severe influenza, as indicated by the need for mechanical ventilation, exhibited a significantly higher risk of developing CFS compared to those who did not require ventilatory support. In contrast, influenza vaccination was associated with a reduced risk of developing CFS. Patients who received the influenza vaccine—either before or following their influenza episode—exhibited a lower incidence of CFS than those who remained unvaccinated. The protective effect of vaccination was not evident in patients with severe influenza requiring ventilation.

**Conclusions:**

Influenza infection is associated with an increased risk of developing CFS. These findings suggest that preventing influenza and mitigating its severity, such as through vaccination, could reduce the burden of CFS in at-risk populations.

## Introduction

Influenza is an acute infectious disease primarily transmitted through the respiratory route, leading to seasonal epidemics and sporadic pandemic outbreaks. The severity of influenza ranges from mild upper respiratory illness to lethal pneumonia. Besides its direct medical impact, influenza also imposes significant economic burdens primarily due to illness-related malaise, restricted activity, and loss of productivity [1, 2]. In the United States alone, seasonal influenza is estimated to account for about $3.2 billion in direct medical costs annually, with indirect costs more than double that amount [2]. Chronic Fatigue Syndrome (CFS) is a disorder characterized by at least six months of unexplained extreme fatigue accompanied by at least four other symptoms, such as impaired memory or concentration, myalgia, multiple joint pain, unrefreshing sleep, and post-exertional malaise [3, 4]. In 2015, the Institute of Medicine (IOM) proposed updated diagnostic criteria that include orthostatic intolerance [5]. These symptoms of CFS significantly reduce a personal’s ability to engage in occupational and social activities. The prevalence of CFS in developed countries is approximately 0.89%, with a higher prevalence in women [6]. In one U.S. cohort, the mean direct medical cost of CFS and intangible socio-economic costs (e.g. lost productivity) were estimated at around USD 14 billion and USD 37 billion per year, respectively [7]. Although the pathophysiology of CFS is not fully understood, it is known that CFS can occur as a sequela of a various infectious diseases, including Epstein–Barr virus (EBV), cytomegalovirus (CMV), *Chlamydia pneumonia*, *Mycoplasma* spp., human herpes virus 6 (HHV6), and Mycobacterium tuberculosis [8–11]. Notably, fatigue is a common symptom during acute influenza infection and is often observed after many other infectious illness, such as COVID-19 [12]. Hence, this raises the question of whether influenza, like other infections, is associated with the development of CFS. Since people usually think that influenza is an acute illness, it is hard to do a long-term follow-up for sequelae in these patients by clinical visits. Prior to our study, a Norwegian cohort study using the national database that infection with the 2009 pandemic influenza A(H1N1) was associated with a subsequent increase in CFS cases, while no increased risk of CFS was observed after influenza vaccination. However, several questions remained unanswered. Dose the severity of an influenza infection influence the likelihood of developing CFS? Does receiving an influenza vaccination protect individuals from CFS, even if they contract influenza? To address these gaps, we designed a large longitudinal cohort study using Taiwan’s NHIRD to explore the relationship between influenza and CFS and to evaluate the effects of influenza severity and vaccination on CFS risk.

## Methods

### Data source

The data for this study were obtained from the National Health Insurance Research Database (NHIRD) of Taiwan, which is maintained by the National Health Research Institutes. The NHIRD is a comprehensive health-related database that covers the entire Taiwanese population under the National Health Insurance program. Diagnoses and medical procedures in NHIRD are coded using the International Classification of Diseases, 9th and 10th Revision, Clinical Modification (ICD-9-CM and ICD-10-CM). Medications are categorized with the Anatomical Therapeutic Chemical (ATC) classification system. To protect privacy in the NHIRD, individual identities are anonymized and encrypted. This study was approved by the Research Ethics Committee of the China Medical University Hospital (CMUH111-REC2-109(CR-1)) and the Institutional Review Board of Mackay Memorial Hospital (16MMHIS074). We implemented measures to reduce potential sources bias, including multivariable adjustments and propensity score matching of cohorts.

### Study population

This cohort study comprises two groups: an influenza (exposure) cohort and a non-influenza (non-exposure) cohort. The influenza cohort included patients who had been diagnosed with influenza (ICD-9-CM 487, or 488; ICD-10-CM J09-J11) [13, 14]. For each patient in the influenza cohort, the date of first influenza diagnosis was defined as the index date. The non-influenza cohort consisted of individuals with no record of influenza and a random date between 2000 and 2018 was assigned as the index date for each of these control individuals.

We applied the following exclusion criteria to both cohorts: (1) age less than 20 or over 100 at the index date; (2) missing data on sex; (3) any recorded diagnosis of the outcome (CFS) before the index date; and (4) any history of certain chronic illnesses before the index date.

The comorbid illnesses leading to exclusion included: cancer (ICD-9-CM: 140–208; ICD-10-CM: C00-C97), sleep apnea (ICD-9-CM: 327.2, 780.51, 780.53, 780.57; ICD-10-CM: G47.3), narcolepsy (ICD-9-CM: 327.0, 327.1; ICD-10-CM: G47.4), depression (ICD-9-CM: 296.2, 296.3, 300.4, 311; ICD-10-CM: F32.0, F32.1, F32.2, F32.3, F32.4, F32.5, F34.1), bipolar affective disorders (ICD-9-CM: 296.4-296.8; ICD-10-CM: F31), schizophrenia (ICD-9-CM: 295; ICD-10-CM: F20), delusional disorders (ICD-9-CM: 297; ICD-10-CM: F22), anorexia and bulimia nervosa (ICD-9-CM: 307.1, 307.51; ICD-10-CM: F500, F501, F502), alcohol or other substance abuse (ICD-9-CM: 291, 303, 305.0, 571.0-571.3, 790.3, V11.3, V79.1; ICD-10-CM: F10, K70, R78.0, Z65.8), systemic lupus erythematosus (SLE) (ICD-9-CM: 710.0; ICD-10-CM: M32), multiple sclerosis (ICD-9-CM: 340; ICD-10-CM: G35), human immunodeficiency virus (HIV) (ICD-9-CM: 042; ICD-10-CM: B20), rheumatoid arthritis (ICD-9-CM: 714; ICD-10-CM: M06.9), and inflammatory bowel disease (ICD-9-CM: 555.0-555.2, 555.9, 556; ICD-10-CM: K50-K51). After exclusions, we used 1:1 propensity score matching to pair each influenza patient with a non-influenzas control based on age, sex, index year, and Charlson Comorbidity Index (CCI) [10, 15, 16].

### Main outcome, comorbidities and treatments

The primary outcome was the incidence of CFS after the index date, defined by a new diagnosis of CFS(ICD-9-CM: 780.7; ICD-10-CM: R53.82, G93.3) between 2000 and 2019 [8–10, 17]. We also examined a range of comorbid conditions present before the index date. These included untreated hypothyroidism (ICD-9-CM: 243, 244; ICD-10-CM: E02, E03, E89.0), diabetes mellitus (DM) (ICD-9-CM: 250; ICD-10-CM: E08-E13), renal disease (ICD-9-CM: 580–589, 593.9; ICD-10-CM: N00-N08, N14-N19, N25-N27, N28.9, N29), insomnia (ICD-9-CM: 780.5, 780.52, 307.42, 327.0; ICD-10-CM: G47.0), anxiety (ICD-9-CM: 300.0, 300.2, 300.3, 308.3, 308.9; ICD-10-CM: F40, F41, F42, F43.0, R45.7), dementia (ICD-9-CM: 290, 294.1, 331.2; ICD-10-CM: F01, F02, F03, F05, G31.1), peptic ulcer (ICD-9-CM: 531, 532, 533; ICD-10-CM: K25, K26, K27), obesity (ICD-9-CM: 278, 783.1; ICD-10-CM: E66.09, E66.1, E66.8, E66.9, E66.01, E66.2, E65, E67.0, E67.1, E67.3, E67.2, E67.8, E68, R63.5), psoriasis (ICD-9-CM: 696; ICD-10-CM: L40), burn (ICD-9-CM: 940–949; ICD-10-CM: T20-T32), gout (ICD-9-CM: 274; ICD-10-CM: M10), dyslipidemia (ICD-9-CM: 272; ICD-10-CM: E71.30, E75.21, E75.22, E75.24, E75.3, E75.5, E75.6, E77, E78.0, E78.1, E78.2, E78.3, E78.4, E78.5, E78.6, E78.70, E78.79, E78.8, E78.9), Sjogren’s (ICD-9-CM: 710.2; ICD-10-CM: M35.0), irritable bowel syndrome (ICD-9-CM: 564.1; ICD-10-CM: K58), hepatitis B virus (HBV) (ICD-9-CM: 070.2, 070.3, V02.61; ICD-10-CM: B16.2, B16.1, B16.0, B16.9, B18.0, B18.1, B19.10, B19.11, Z22.51), hepatitis C virus (HCV) (ICD-9-CM: 070.41, 070.44, 070.51, 070.54, 070.70, 070.71, V02.62; ICD-10-CM: B17.10, B17.11, B19.20, B19.21, B18.2, Z22.52), and fibromyalgia (ICD-9-CM: 729.1; ICD-10-CM: M79.7). Additionally, we evaluated two key interventions/ indicators during the course of influenza illness: influenza vaccination (healthcare procedure code: A2001C) and mechanical ventilation support (procedure codes: 57023B, 57001B).

### Statistical analyses

We conducted all analyses using a longitudinal cohort design to compare outcomes between the influenza and non-influenza groups. Baseline characteristics were summarized using descriptive statistics, such as mean, standard deviation, and proportion, and the balance between cohorts after matching was assessed by calculating standardized mean differences (SMD). An SMD < 0.1 was considered to indicate negligible difference in a covariate between the two cohorts. Time-to-event analyses were performed to assess the risk of CFS. We used Kaplan-Meier (KM) curves to estimate the cumulative incidence of CFS over time in each cohort and compared the survival curves using the log-rank test. Cox proportional hazards regression models were then applied to estimate hazard ratios (HRs) and 95% confidence intervals for the development of CFS, with the influenza cohort compared to the non-influenza cohort [8–10]. The Cox models adjusted for potential confounders including age, sex, and baseline comorbidities. We report adjusted HRs (aHRs) with two-tailed p-values, considering *p* < 0.05 as statistically significant.

To investigate the effects of influenza severity and vaccination, we performed subgroup and interaction analyses. We stratified patients by age group, sex, CCI score, and presence or absence of any baseline comorbidity to see if the association between influenza and CFS held across these subgroups. We also stratified the influenza cohort based on whether patients received an influenza vaccine, before or after the index date, and whether they required mechanical ventilation during the acute illness. Cox models were used in these strata to estimate CFS risk in vaccinated vs. unvaccinated, and in ventilated vs. non-ventilated patients. All analyses were executed using SAS and R statistical software.

### Literature search

A systematic literature search was conduct for relevant articles until March 1, 2025, in PubMed, EMBASE and Cochrane Collaboration using the following terms: Influenza and Chronic fatigue syndrome. No restrictions on language or study type were applied.

## Results

**Fig. 1:**
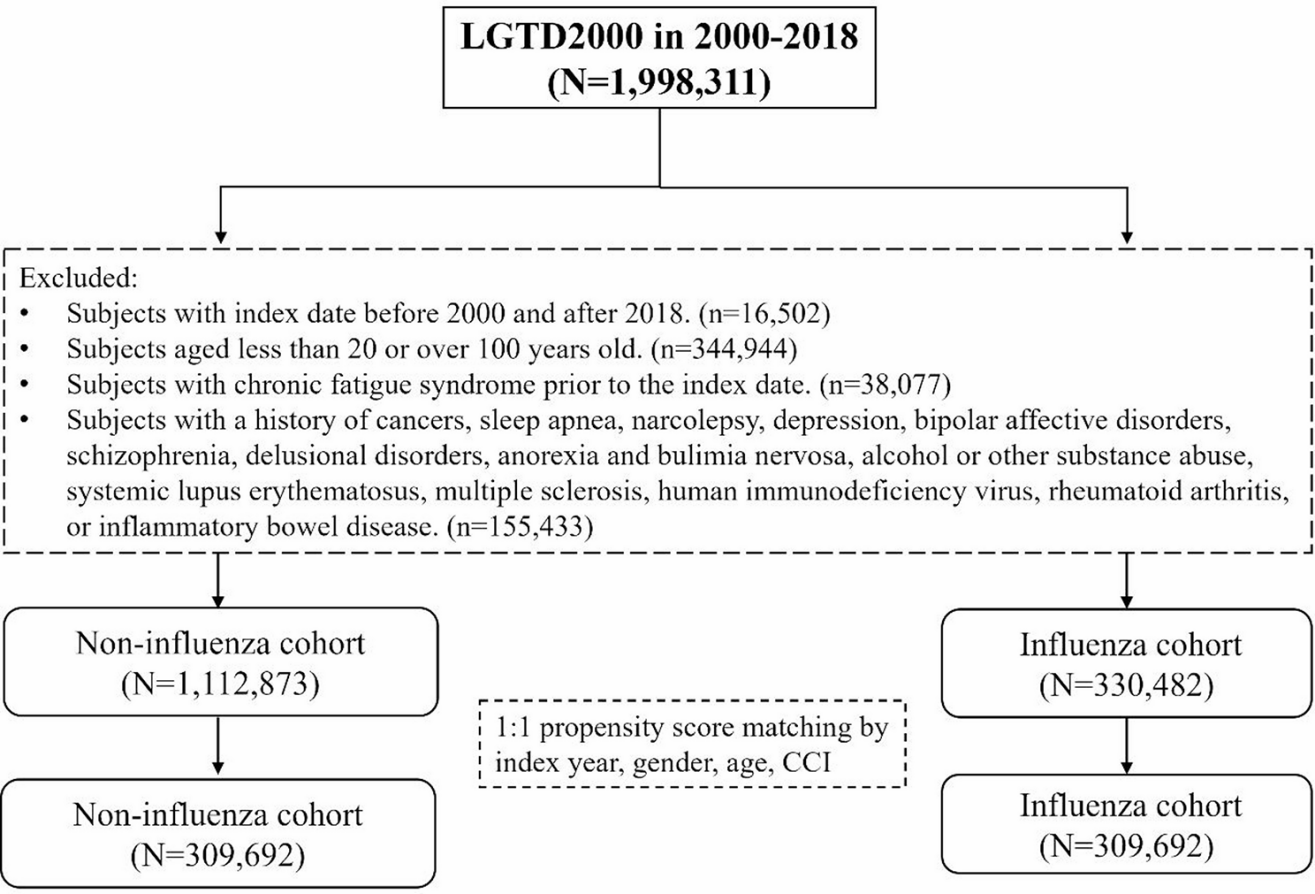
The selection process of the participants. * LGTD2000 = Longitudinal Generation Tracking Database, a derivative of the extensive National Health Insurance Research Database (NHIRD)

After applying exclusion criteria and propensity score matching, we identified 309,692 patients in the influenza cohort and 309,692 patients in the non-influenza cohor process t. Table 1 summarizes the baseline characteristics of the two matched cohorts, and Fig. 1 illustrates the patient selection flow chart. The matching procedure was successful: there were no significant differences between the cohorts in key baseline variables (all SMDs < 0.1). The age distributions were similar in both groups, with approximately 48% of patients aged 20–39 years, 38% aged 40–64 years, and 13% aged 65–100 years. The sex ratio was also balanced, namely, about 45% male and 55% female in each cohort. The mean follow-up duration was 12.04 ± 5.50 years for the influenza cohort and 11.26 ± 5.72 years for the non-influenza cohort. The influenza cohort had a markedly higher incidence of CFS compared to the non-influenza cohort. As shown in Table 2, having had an influenza infection was associated with a significantly increased risk of developing CFS subsequently (aHR = 1.51, 95% CI: 1.48–1.55, compared to no influenza). This elevated risk was evident across all demographic subgroups. In particular, older age amplified the risk of CFS following influenza: individuals aged 40–64 years had about a 1.31-fold higher risk (95% CI: 1.28–1.35), and those aged ≥ 65 years had roughly a 1.91-fold higher risk (95% CI: 1.85–1.97), compared to the 20–39 year age group. Interestingly, male sex was associated with a slightly lower risk of CFS than female sex (aHR = 0.91, 95% CI: 0.89–0.93). As shown in Table 2, having had an influenza infection was associated with a significantly higher risk of developing CFS subsequently (aHR = 1.51, 95% CI: 1.48–1.55, compared to no influenza). The elevated risk was evident across all demographic subgroups. In particular, older age amplified the risk of CFS following influenza: individuals aged 40–64 years had about a 1.31-fold higher risk (95% CI: 1.28–1.35), and those aged ≥ 65 years had roughly a 1.91-fold higher risk (95% CI: 1.85–1.97), compared to the 20–39 years age group. Interestingly, male sex was associated with a slightly lower risk of CFS than female sex (aHR = 0.91, 95% CI: 0.89–0.93). Aside from age and sex, several pre-existing conditions were independent risk factors for CFS. Patients with certain comorbidities experienced significantly higher rates of CFS than those without those conditions. Notably, a history of insomnia (aHR = 1.40, 95% CI: 1.36–1.44), anxiety (aHR = 1.22, 95% CI: 1.17–1.26), peptic ulcer disease (aHR = 1.18, 95% CI: 1.14–1.21), gout (aHR = 1.18, 95% CI: 1.14–1.23), dyslipidemia (aHR = 1.06, 95% CI: 1.03–1.10), irritable bowel syndrome (aHR = 1.14, 95% CI: 1.08–1.20), chronic HBV infection (aHR = 1.13, 95% CI: 1.05–1.21), HCV infection (aHR = 1.39, 95% CI: 1.25–1.55), and fibromyalgia (aHR = 1.26, 95% CI: 1.22–1.29) were all associated with an increased risk of developing CFS, compared to patients without these conditions.

**Table 1:** Demographic characteristics for individuals with and without influenza

	Influenza				
	No		Yes		
	*N* = 309,692		*N* = 309,692		
	n	%	n	%	*SMD*
**Age**					
20–39	150,487	48.59	147,580	47.65	0.019
40–64	116,863	37.74	119,088	38.45	0.015
65–100	42,342	13.67	43,024	13.89	0.006
Mean ± SD	43.22	16.51	43.53	16.43	0.019
**Gender**					0.047
Women	174,685	56.41	167,377	54.05	
Men	135,007	43.59	142,315	45.95	
**CCI**					
0	295,778	95.51	295,738	95.49	0.001
1	8386	2.71	8420	2.72	0.001
≥ 2	5528	1.78	5534	1.79	< 0.001
**Comorbidity**					
Untreated hypothyroidism	1343	0.43	1588	0.51	0.012
DM	4632	1.50	4333	1.40	0.008
Renal disease	1248	0.40	892	0.29	0.020
Insomnia	34,057	11.00	46,100	14.89	0.116
Anxiety	17,996	5.81	24,023	7.76	0.077
Dementia	245	0.08	171	0.06	0.009
Peptic ulcer	43,031	13.89	54,568	17.62	0.102
Obesity	1696	0.55	2020	0.65	0.014
Psoriasis	2715	0.88	2955	0.95	0.008
Burn	73	0.02	66	0.02	0.002
Gout	18,270	5.90	21,371	6.90	0.041
Dyslipidemia	30,841	9.96	35,615	11.50	0.050
Sjogren’s	88	0.03	113	0.04	0.004
Irritable bowel syndrome	10,023	3.24	14,049	4.54	0.067
HBV	6313	2.04	7638	2.47	0.029
HCV	1769	0.57	2164	0.70	0.016
Fibromyalgia	40,708	13.14	54,894	17.73	0.127
**Followup (years)**					
Mean ± SD	11.26 ± 5.72		12.04 ± 5.5		0.139

**Table 2:** Risk factor analyses for CFS among all study individuals

	Event	PY	Rate ^†^		Crude HR			Adjusted HR ^#^	
				HR	(95% CI)	p-value	aHR	(95% CI)	p-value
**Influenza**									
No	13,245	3,485,683	3.80	1.00	(reference)	-	1.00	(reference)	-
Yes	22,601	3,727,563	6.06	1.6	(1.57, 1.64)***	< 0.001	1.51	(1.48, 1.55)***	< 0.001
**Age**									
20–39	14,450	3,737,934	3.87	1.00	(reference)	-	1.00	(reference)	-
40–64	15,325	2,762,990	5.55	1.45	(1.41, 1.48)***	< 0.001	1.31	(1.28, 1.35)***	< 0.001
65–100	6071	712,321	8.52	2.24	(2.18, 2.31)***	< 0.001	1.91	(1.85, 1.97)***	< 0.001
**Gender**									
Women	21,327	4,073,131	5.24	1.00	(reference)	-	1.00	(reference)	-
Men	14,519	3,140,115	4.62	0.89	(0.87, 0.91)***	< 0.001	0.91	(0.89, 0.93)***	< 0.001
**CCI**									
0	34,254	7,013,836	4.88	1.00	(reference)	-	1.00	(reference)	-
1	1055	133,936	7.88	1.6	(1.5, 1.7)***	< 0.001	1.04	(0.97, 1.11)	0.2447
≥ 2	537	65,474	8.20	1.72	(1.58, 1.87)***	< 0.001	1.03	(0.93, 1.14)	0.582
**Comorbidity**									
**Untreated hypothyroidism**									
No	35,667	7,188,151	4.96	1.00	(reference)	-	1.00	(reference)	-
Yes	179	25,094	7.13	1.43	(1.24, 1.66)***	< 0.001	1.04	(0.89, 1.2)	0.6365
DM									
No	35,352	7,154,080	4.94	1.00	(reference)	-	1.00	(reference)	-
Yes	494	59,165	8.35	1.7	(1.56, 1.86)***	< 0.001	0.98	(0.88, 1.09)	0.6785
**Renal disease**									
No	35,798	7,203,160	4.97	1.00	(reference)	-	1.00	(reference)	-
Yes	48	10,085	4.76	1	(0.75, 1.33)	> 0.999	0.58	(0.43, 0.79)***	< 0.001
**Insomnia**									
No	29,757	6,507,528	4.57	1.00	(reference)	-	1.00	(reference)	-
Yes	6089	705,718	8.63	1.86	(1.81, 1.91)***	< 0.001	1.4	(1.36, 1.44)***	< 0.001
**Anxiety**									
No	32,447	6,831,568	4.75	1.00	(reference)	-	1.00	(reference)	-
Yes	3399	381,677	8.91	1.85	(1.79, 1.92)***	< 0.001	1.22	(1.17, 1.26)***	< 0.001
**Dementia**									
No	35,833	7,211,826	4.97	1.00	(reference)	-	1.00	(reference)	-
Yes	13	1419	9.16	2.09	(1.21, 3.59)**	0.0079	1	(0.58, 1.73)	0.9956
**Peptic ulcer**									
No	28,991	6,302,685	4.60	1.00	(reference)	-	1.00	(reference)	-
Yes	6855	910,561	7.53	1.62	(1.57, 1.66)***	< 0.001	1.18	(1.14, 1.21)***	< 0.001
**Obesity**									
No	35,660	7,181,224	4.97	1.00	(reference)	-	1.00	(reference)	-
Yes	186	32,022	5.81	1.15	(1, 1.33)	0.0547	0.93	(0.8, 1.07)	0.2932
**Psoriasis**									
No	35,595	7,164,474	4.97	1.00	(reference)	-	1.00	(reference)	-
Yes	251	48,771	5.15	1.03	(0.91, 1.16)	0.6886	0.96	(0.85, 1.09)	0.5543
**Burn**									
No	35,837	7,211,804	4.97	1.00	(reference)	-	1.00	(reference)	-
Yes	9	1442	6.24	1.25	(0.65, 2.41)	0.4963	1.31	(0.68, 2.51)	0.4241
**Gout**									
No	33,034	6,849,479	4.82	1.00	(reference)	-	1.00	(reference)	-
Yes	2812	363,767	7.73	1.59	(1.53, 1.65)***	< 0.001	1.18	(1.14, 1.23)***	< 0.001
**Dyslipidemia**									
No	31,234	6,628,926	4.71	1.00	(reference)	-	1.00	(reference)	-
Yes	4612	584,320	7.89	1.66	(1.61, 1.72)***	< 0.001	1.06	(1.03, 1.1)***	< 0.001
**Sjogren’s**									
No	35,841	7,211,807	4.97	1.00	(reference)	-	1.00	(reference)	-
Yes	5	1439	3.48	0.7	(0.29, 1.69)	0.4283	0.43	(0.18, 1.04)	0.0626
**Irritable bowel syndrome**									
No	34,084	6,994,716	4.87	1.00	(reference)	-	1.00	(reference)	-
Yes	1762	218,529	8.06	1.63	(1.56, 1.72)***	< 0.001	1.14	(1.08, 1.2)***	< 0.001
**HBV**									
No	35,028	7,088,014	4.94	1.00	(reference)	-	1.00	(reference)	-
Yes	818	125,231	6.53	1.3	(1.22, 1.4)***	< 0.001	1.13	(1.05, 1.21)***	< 0.001
**HCV**									
No	35,506	7,180,779	4.94	1.00	(reference)	-	1.00	(reference)	-
Yes	340	32,466	10.47	2.11	(1.9, 2.35)***	< 0.001	1.39	(1.25, 1.55)***	< 0.001
**Fibromyalgia**									
No	29,267	6,334,830	4.62	1.00	(reference)	-	1.00	(reference)	-
Yes	6579	878,416	7.49	1.59	(1.55, 1.63)***	< 0.001	1.26	(1.22, 1.29)***	< 0.001

PY: Person-Year, IR: Incidence rate, per 1000 persons/years; HR: Hazard ratio; CI: confidence interval; Adjusted HR: adjusted for age, sex, and comorbidities in Cox proportional hazards regression. *p < 0.05, **p < 0.01, ***p < 0.001

**Fig. 2:**
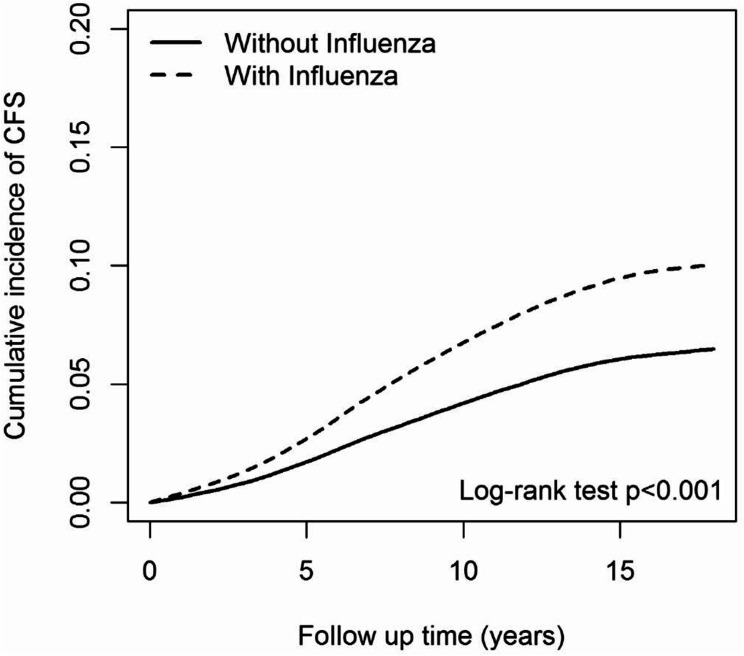
Cumulative incidence of CFS compared between patients with and without influenza using the Kaplan–Meier method

The Kaplan-Meier curves for CFS-free survival (cumulative incidence of CFS) are shown in Fig. 2. Consistent with the Cox regression results, the influenza cohort demonstrated a significantly higher risk cumulative incidence of CFS over time than the non-influenza cohort (log-rank test *p* < 0.001). By the end of the follow-up period, the probability of remaining CFS-free was substantially lower in the influenza group, underscoring the long-term risk posed by influenza infection.

We performed additional analyses to explore the influence of specific factors on CFS risk in the influenza cohort, shown in Table 3. These stratified analyses indicated that the association between influenza and CFS persisted in every examined subgroup. The increased risk of CFS in influenza patients was observed irrespective of age group, specifically, young, middle-aged, or elderly, sex (in both women and men), baseline comorbidity burden as exemplified by patients with low CCI vs. high CCI, and presence of any specific comorbidity, in other words, both in patients with and without pre-existing comorbid conditions. In other words, influenza was consistently a risk factor for CFS across all these categories, with adjusted hazard ratios generally in the 1.4–1.6 range in each subgroup.

**Table 3:** Incidences and hazard ratios of CFS for individuals with and without influenza by age, gender, and comorbidity

	Influenza											
	No			Yes				Crude HR			Adjusted HR	
	Event	PY	Rate	Event	PY	Rate	HR	(95% CI)	p-value	aHR	(95% CI)	p-value
Age												
20–39	5830	1,871,296	3.12	8620	1,866,638	4.62	1.49	(1.44, 1.54)***	< 0.001	1.43	(1.38, 1.48)***	< 0.001
40–64	5489	1,316,619	4.17	9836	1,446,371	6.80	1.64	(1.59, 1.7)***	< 0.001	1.58	(1.53, 1.64)***	< 0.001
65–100	1926	297,768	6.47	4145	414,554	10.00	1.51	(1.43, 1.59)***	< 0.001	1.49	(1.41, 1.57)***	< 0.001
Gender												
Women	8430	2,046,003	4.12	12,897	2,027,128	6.36	1.55	(1.51, 1.59)***	< 0.001	1.45	(1.41, 1.49)***	< 0.001
Men	4815	1,439,680	3.34	9704	1,700,435	5.71	1.71	(1.66, 1.77)***	< 0.001	1.62	(1.56, 1.68)***	< 0.001
CCI												
0	12,751	3,398,414	3.75	21,503	3,615,422	5.95	1.59	(1.56, 1.63)***	< 0.001	1.5	(1.47, 1.54)***	< 0.001
1	341	60,521	5.63	714	73,415	9.73	1.74	(1.53, 1.98)***	< 0.001	1.64	(1.44, 1.87)***	< 0.001
≥ 2	153	26,748	5.72	384	38,726	9.92	1.77	(1.47, 2.14)***	< 0.001	1.62	(1.34, 1.95)***	< 0.001
Comorbidity												
No	7170	2,371,608	3.02	11,447	2,334,218	4.90	1.63	(1.58, 1.68)***	< 0.001	1.57	(1.53, 1.62)***	< 0.001
Yes	6075	1,114,075	5.45	11,154	1,393,345	8.01	1.47	(1.42, 1.51)***	< 0.001	1.42	(1.38, 1.47)***	< 0.001

PY: Person-Year, IR: Incidence rate, per 1000 persons/years; HR: Hazard ratio; CI: confidence interval; Adjusted HR: adjusted for age, sex, and comorbidities in Cox proportional hazards regression. *p < 0.05, **p < 0.01, ***p < 0.001

It was observed that patients with influenza had a significantly higher risk of developing CFS compared to those in the control group, irrespective of age [age 20–39 (aHR = 1.43, 95% CI = 1.38–3.148), age 40–64 (aHR = 1.5, 95% CI = 1.53–8.164), age 65–100 (aHR = 1.4, 95% CI = 1.419–9.157)], sex [women (aHR = 1.4, 95% CI = 1.41–5.149), men (aHR = 1.6, 95% CI = 1.56–2.168)], CCI levels [CCI = 0 (aHR = 1.5, 95% CI = 1.47–1.54), CCI = 1 (aHR = 1.6, 95% CI = 1.44–4.187), CCI ≥ 2 (aHR = 1.6, 95% CI = 1.34–2.195)], and comorbidities [with comorbidities (aHR = 1.5, 95% CI = 1.53–7.162), without comorbidities (aHR = 1.4, 95% CI = 1.38–2.147)].

Next, we examined the effects of influenza vaccination and disease severity on the risk of CFS, as summarized in Table 4 − 1, 4 − 2, and 4 − 3. We found that influenza vaccination was associated with a protective effect against CFS. Patients who received an influenza vaccine, either prior to their influenza infection or afterward in the same influenza season, had a lower risk of developing CFS compared to those who were not vaccinated. This pattern held true even when considering the timing of vaccination relative to the index date. Furthermore, when we focused on patients who did not require mechanical ventilation during their influenza illness, namely, those with relatively milder influenza cases, the vaccinated individuals had a significantly lower incidence of CFS than the unvaccinated individuals.

**Table 4:** 1. Comparison of incidence and hazard ratio of chronic fatigue syndrome between influenza patients with and without vaccination

	Event	PY	Rate		Crude HR			Adjusted HR ^#^	
				HR	(95% CI)	p-value	aHR	(95% CI)	p-value
Vaccination									
No	22,452	3,701,702	6.07	1.00	(reference)	-	1.00	(reference)	-
Yes	149	25,861	5.76	1.09	(0.93, 1.28)	0.2918	0.54	(0.45, 0.63)***	< 0.001

PY: Person-Year, IR: Incidence rate, per 1000 persons/years; HR: Hazard ratio; CI: confidence interval; Adjusted HR: adjusted for age, sex, and comorbidities in Cox proportional hazards regression. *p < 0.05, **p < 0.01, ***p < 0.001

**Table 4:** 2. Comparison of incidence and hazard ratio of chronic fatigue syndrome between influenza patients with mechanical ventilation

	Event	PY	Rate		Crude HR			Adjusted HR ^#^	
				HR	(95% CI)	p-value	aHR	(95% CI)	p-value
Vaccination									
No	3029	328,472	9.22	1.00	(reference)	-	1.00	(reference)	-
Vaccinations (both)	12	1552	7.73	1.1	(0.62, 1.94)	0.7435	0.6	(0.34, 1.07)	0.0852
Only vaccination before influenzas	4	661	6.05	0.84	(0.32, 2.25)	0.7329	0.51	(0.19, 1.35)	0.1743
Only vaccination after influenzas	24	1861	12.90	1.68	(1.12, 2.51)*	0.0117	1.07	(0.71, 1.6)	0.756

PY: Person-Year, IR: Incidence rate, per 1000 persons/years; HR: Hazard ratio; CI: confidence interval; Adjusted HR: adjusted for age, sex, and comorbidities in Cox proportional hazards regression. *p < 0.05, **p < 0.01, ***p < 0.001

**Table 4:** 3. Comparison of incidence and hazard ratio of chronic fatigue syndrome between influenza patients without mechanical ventilation

	Event	PY	Rate		Crude HR			Adjusted HR ^#^	
				HR	(95% CI)	p-value	aHR	(95% CI)	p-value
Vaccination									
No	19,423	3,373,229	5.76	1.00	(reference)	-	1.00	(reference)	-
Vaccinations (both)	38	7788	4.88	0.96	(0.7, 1.32)	0.8179	0.45	(0.33, 0.62)***	< 0.001
Only vaccination before influenzas	17	3451	4.93	0.94	(0.58, 1.51)	0.7975	0.46	(0.29, 0.75)**	0.0016
Only vaccination after influenzas	54	10,547	5.12	1.01	(0.78, 1.32)	0.9204	0.54	(0.41, 0.71)***	< 0.001

PY: Person-Year, IR: Incidence rate, per 1000 persons/years; HR: Hazard ratio; CI: confidence interval; Adjusted HR: adjusted for age, sex, and comorbidities in Cox proportional hazards regression. *p < 0.05, **p < 0.01, ***p < 0.001

Finally, we assessed the impact of mechanical ventilation, which serves as a proxy for severe influenza infection, such as pneumonia or respiratory failure. Table 5 shows that among patients in the influenza cohort, those who needed mechanical ventilation had a higher likelihood of developing CFS compared to those who did not need ventilatory support. In other words, severe influenza was associated with increased CFS susceptibility. Notably, in the subgroup of patients who did require mechanical ventilation, prior influenza vaccination did not seem to confer a noticeable reduction in CFS risk, in contrast to the clear benefits of vaccination observed in non-ventilated patients.

**Table 5:** Comparison of incidence and hazard ratio of chronic fatigue syndrome between influenza patients with or without mechanical ventilation

	Event	PY	Rate		Crude HR			Adjusted HR	
				HR	(95% CI)	p-value	aHR	(95% CI)	p-value
Mechanical ventilation									
No	31,158	6,597,877	5.75	4.72	1.00	(reference)	1.00	(reference)	-
Yes	4688	615,369	9.23	7.62	1.62 (1.58, 1.68)***	< 0.001	1.29	(1.25, 1.34)***	< 0.001

PY: Person-Year, IR: Incidence rate, per 1000 persons/years; HR: Hazard ratio; CI: confidence interval; Adjusted HR: adjusted for age, sex, and comorbidities in Cox proportional hazards regression. *p < 0.05, **p < 0.01, ***p < 0.001

## Discussion

In this nationwide, population-based longitudinal cohort study, we found that individuals who contracted influenza had a higher risk of subsequently developing CFS than those who did not have influenza. Our results also suggest that influenza vaccination can reduce the risk of CFS. Importantly, the protective effect of vaccination was most evident in patients with less severe influenza illness, those who did not require mechanical ventilation. Among such patients, receiving an influenza vaccine—whether before or after the influenza infection—was associated with a lower incidence of CFS compared to not being vaccinated. On the other hand, in patients who experienced severe influenza requiring mechanical ventilation, we did not observe a significant reduction in CFS risk from vaccination. This implies that the severity of the influenza infection plays a role in modulating CFS risk: patients with severe influenza were more likely to develop CFS than those with milder influenza, and in these severe cases, the benefit of vaccination in preventing CFS was not apparent.

Our findings provided substantial evidence to the limited existing literature on post-influenza CFS. Previously, a Norwegian registry-based study focusing on the 2009 H1N1 influenza pandemic reported that patients infected with the pandemic influenza strain had an increased risk of developing CFS/ME in the following three years. In contrast, no increased CFS risk was observed among those who received an H1N1 influenza vaccination [18]. However, there are still some questions left unanswered. We extend those observations in several ways. First, our study reveals that the association between influenza and CFS is not confined to the 2009 A(H1N1) pandemic strain; rather, influenza infection in general- across multiple seasons and virus subtypes over 17 years- is associated with an elevated risk of CFS. Second, we provide evidence that the severity of influenza infection may influence the likelihood of CFS. As far as we are aware, this represents the first study to demonstrate that patients with life-threatening influenza, as indicated by the need for ICU-level care or mechanical ventilation, have a higher propensity for subsequent CFS compared to those with less severe influenza. Third, we found that influenza vaccination is associated with a reduced risk of CFS, even among individuals who later contract influenza. This suggests that vaccination may confer some protection or mitigation beyond just preventing influenza infection—it may also attenuate the long-term sequelae such as CFS if infection occurs.

We also observed that older age was a significant risk factor for developing CFS after influenza. In our cohort, the risk of CFS was progressively higher in middle-aged and elderly patients compared to younger adults. For example, patients aged 40–64 had about a 1.31-fold greater risk, and those aged 65 and above had roughly a 1.5-fold greater risk of CFS, relative to the 20–39 age group (Table 2). This age-related pattern underscores the importance of preventive measures against influenza in older populations. Influenza vaccination is especially crucial for the elderly, not only because it may avert the development of chronic complications such as CFS. Influenza vaccination is well known to have multiple benefits: it helps prevent influenza infection, decreases the severity and mortality of influenza in those who become infected, and reduces the risk of transmitting the virus to others, particularly vulnerable individuals [19–21]. Moreover, seasonal influenza vaccination is a cost-effective public health strategy, yielding economic benefits in targeted groups such as healthcare workers and the elderly by averting medical visits, hospitalizations, and days of lost productivity [22, 23]. 

Our study suggests a new potential benefit of vaccination — the prevention of CFS — which could further encourage influenza vaccination, especially in older adults. Despite the known benefits, vaccine hesitancy persists in some segments of the population. Concerns often center around side effects and the misconception that the vaccine might cause illness. Influenza vaccines can indeed cause transient side effects in some people, such as injection-site pain, low-grade fever, headache, or myalgia. These post-vaccination symptoms are generally mild and short-lived and can be explained by the acute immune response triggered by the vaccine. For instance, proinflammatory cytokines like TNF-α and macrophage migration inhibitory factor (MIF) are elevated on the day after influenza vaccination, correlating with these minor symptoms [24]. However, the immune activation from a vaccine is much limited in scope and duration compared to that induced by an actual influenza infection [25]. Given that an actual influenza infection can lead not only to immediate illness but also to prolonged sequelae such as CFS, the short-term discomfort of vaccination is a small price to pay for potentially avoiding long-term complications [26, 27]. Therefore, we should continue to strongly encourage influenza vaccination, particularly for individuals at high risk, as a means of preventing CFS and other post-infectious syndromes.

The mechanisms linking influenza infection to the development of CFS are still not fully understood, but several hypotheses have been proposed. One theory posits that certain individuals have a genetic predisposition that causes their B -cell to be prone to autoreactivity upon minor external stimulation. In such individuals, a decisive triggering like a significant infection could initiate an autoimmune response against self-antigens, possibly including components of energy metabolism or mitochondria function. This autoimmune process could manifest clinically as profound fatigue and related symptoms of CFS [28]. Another line of investigation involves cellular aging and immune senescence: recent studies have shown that patients with CFS tend to have significantly shorter telomeres in their leukocytes compared to healthy controls [29]. Telomere length is also associated with immune responses in the context of influenza. For example, among older adults, those with longer telomeres of B cells have been observed to mount better antibody responses to influenza vaccination [30, 31]., and CD8 + T cells specific to influenza, such as those targeting the conserved M1 protein, with longer telomeres can expand more robustly upon activation [30]. 

These findings imply that individuals with longer telomere in immune cells more are resilient and less likely to influenza and its complications, whereas shorter telomeres, as seen in CFS patients, could signify a reduced capacity to cope with infections and an increased susceptibility to prolonged post-infectious fatigue.

Our analysis of patients with severe influenza provides additional clues about the possible pathophysiology linking influenza to CFS. Patients who required mechanical ventilation for influenza had a higher subsequent risk of CFS shown in Table 5, suggesting that an intense inflammatory response or tissue injury during severe influenza illness might predispose to CFS. Severe influenza is known to trigger a “cytokine storm” or an exaggerated inflammatory response. Indeed, prior studies have documented those patients with severe influenza, e.g., those hospitalized in ICU, have significantly elevated levels of numerous cytokines and chemokines, including IL-6, IL-10, IL-15, CXCL10 (IP-10), soluble IL-2 receptor (sIL-2R), hepatocyte growth factor (HGF), ST2, and CXCL9 (MIG), compared to patients with mild influenza [32]. In contrast, research on CFS has identified a cytokine signature associated with the severity of CFS: higher circulating levels of CCL11 (Eotaxin-1), CXCL1 (GROα), CXCL10 (IP-10), IFN-γ, IL-4, IL-5, IL-7, IL-12p70, IL-13, IL-17 F, leptin, G-CSF, GM-CSF, LIF, NGF, SCF, and TGF-α have been reported in more severe CFS cases [27]. Interestingly, the only cytokine overlapping between the severe influenza profile and the CFS profile is CXCL10 (IP-10), which is elevated in both conditions. Meanwhile, CXCL9 (MIG), which is strongly elevated in severe influenza, isinversely correlated with the duration of fatigue in CFS; in other words, higher CXCL9 is linked to shorter fatigue duration. These immunological findings hint at complex interactions: a severe influenza infection causes a broad immune activation that might set the stage for CFS in susceptible individuals, but the specific immune mediators driving persistent fatigue may differ from those driving acute severity. Further studies are needed to delineate how the acute inflammatory milieu of severe influenza transitions into the chronic immune dysregulation observed in CFS.

In addition, in Table 4**− 1**, we found that receiving an influenza vaccination decreases the risk of developing CFS. Moreover, it is interesting that people with milder diseases can also benefit from the CFS-lowering effect of vaccination even after they have had influenza (Table 4**− 3**). We also explored why the benefit of vaccination was not evident in the most severe influenza cases. Our data showed that influenza vaccination did not significantly reduce CFS incidence in patients who required mechanical ventilation, namely, those with very severe disease, even though it reduced the risk of CFS in non-ventilated patients. One possible explanation is that once influenza reaches a life-threatening severity, the subsequent risk of long-term fatigue is dominated by factors inherent to critical illness, which vaccination cannot quickly mitigate. Supporting this idea, past studies have noted that more than half of survivors of intensive care (ICU) treatment report chronic fatigue lasting from 6 to 70 months after ICU discharge [33] This post-ICU fatigue syndrome has a multifactorial origin, attributed to issues like profound muscle deconditioning, malnutrition, protracted rehabilitation efforts, and sleep disturbances that often accompany prolonged critical illness and recovery This post-ICU fatigue syndrome has a multifactorial origin, attributed to issues like profound muscle deconditioning, malnutrition, protracted rehabilitation efforts, and sleep disturbances that often accompany prolonged critical illness and recovery [34]. Such factors could similarly affect patients who survive severe influenza, potentially overshadowing any protective effect that prior vaccination might have conferred against developing CFS. In other words, once a patient experiences a severe influenza infection necessitating mechanical ventilation, the physiological stress and aftermath of critical illness might overwhelm the moderating influence of vaccination on long-term outcomes.

In summary, we have explored several potential mechanisms connecting influenza infection to CFS, including immunological and cellular pathways. However, the pathophysiology of CFS is complex and remains only partially understood. Many questions—such as why only a subset of individuals develop CFS after infections and how exactly vaccination modulates long-term outcomes—remain unanswered and warrant further investigation [35].

### Strengths and limitations of this study

Our study has several limitations. First, detailed clinical information on patients was not available in the NHIRD–to be specific, this includes symptom severity, such as the exact severity of fatigue or other CFS - symptoms, occupation, and virus genotype is not available. Second, diagnoses of influenza in the database were made by physicians and not all were confirmed by laboratory tests; during influenza epidemics some patients are diagnosed based on clinical presentation alone, which could lead to misclassification (though the extent of this is unknown). Third, while antiviral medications (oseltamivir or zanamivir) are provided free of charge to confirmed influenza patients in Taiwan’s health system, some patients may have obtained other antivirals like peramivir through out-of-pocket purchase. The NHIRD does not capture medications that are not covered by insurance, so we could not account for the use of self-paid antivirals, which might influence outcomes.

Despite these limitations, our study also has notable strengths. The NHIRD covers approximately 99.9% of Taiwan’s population, providing a virtually population-wide sample and minimizing selection bias. The large sample size and long follow-up enhance the power to detect associations and study rare outcomes like CFS. Moreover, the NHIRD has been validated for research purposes [36] and is regularly updated and audited to ensure data integrity and accuracy. These features of the database reduce the potential for data errors and biases, lending credibility to our findings.

## Conclusion

In this large cohort study, influenza infection was associated with an increased risk of subsequent CFS. As far as we are aware, no prior research has demonstrated that the severity of influenza can significantly affect the risk of developing CFS, with severe influenza leading to a higher risk. We also found that influenza vaccination is associated with a protective effect against CFS. These findings suggest that individuals at risk of CFS, for example, those with underlying conditions or older age, should consider influenza vaccination as a preventive measure to reduce their risk of developing CFS after an influenza infection. Overall, our study provides new insights into the long-term consequences of influenza and highlights the importance of influenza prevention and management in reducing the burden of CFS.

## Abbreviations

CCI: Charleson Comorbidity Index
CFS: Chronic fatigue syndrome
CMV: Cytomegalovirus
DM: Diabetes mellitus
EBV: Epstein–Barr virus
HBV: Hepatitis B virus
HCV: Hepatitis C virus
HHV6: Human herpes virus 6
NHIRD: National Health Insurance Research Database
SARS: Severe acute respiratory syndrome
